# Anæsthesia by Rapid Respiration

**Published:** 1878-07

**Authors:** A. H. R. Guiley


### ARTICLE VII.

### *Anæsthesia by Rapid Respiration.*

Dr. A. H. R. Guiley writes to the *Medical and Surgical Reporter* as follows:

The writer's thesis, under the above title (written July 6th, 1876,) was awarded the "J. M. Toner gold medal," at the commencement of Jefferson Medical College, Philadelphia, March, 1877. "Bonwill's method" was carefully investigated, and the results and conclusions were given in the paper alluded to, from which the following remarks are principally derived.

Dr. Bonwill's method is described in *Pennsylvania Journal of Dental Science*, for February, 1876. He found that air, "drawn into the lungs, in quantities three or four times as great as required by the body," was capable of producing anæsthesia sufficiently profound to render minor surgical operations painless. Dr. Bonwill could only account for the anæsthesia by giving the credit of its production to a super-oxygenation, or a surplus of nitrogen, or to a mixture of the two. The author concluded, from numerous experiments, that—

1st. Breathing full, at ninety to the minute, will produce anæsthesia of the surface of the body in less than five minutes, so that a pin or needle thrust half an inch into a limb is not felt. (This is Bonwill's method.)

2d. This anæsthesia is not due to "a surplus of oxygen or of nitrogen, or of the two combined," but follows from the rapidity of the breathing, according to laws certainly known since the beginning of this century.

3d. The rapidity of the breathing induces the anæsthesia by the following sequence:—

(*a*) By their over-action the blood of the body is determined toward the lungs. [Any muscle or organ violently exercised for a short time, receives a surplus of blood; a well-known fact.]

(*b*) The blood having assumed this centripetal determination leaves the surface of the body.

(*c*) When the surface of the body is deprived of its blood, superficial anæsthesia supervenes. Wardrof, in 1819, produced anæsthesia for surgical operations by venesection until the surface was combletely blanched. (See Gross' Syst. Surg., Vol. 1, p. 575. Sec. ed.) Within a few months superficial anæsthesia has been produced by the application of Esmarch's bandage, rendering the parts anæmic.

3

In fact (*c*) might be made more general, as—

(*d*) A part completely deprived of communication with the trunk by blood supply, or return, will be anæsthetized. But (*d*) is not necessary, from the fact that for an amputation, the surgeon will depend on an established anæsthetic. The complete withdrawal of the blood from a part involves an anæmia of the nerves, which probably is the direct cause of the anæsthesia.

With these facts before him the experimenter can account for the congestion of the lungs; the tingling first felt at the extremities and extending toward the central organs; the labored action of the heart; the clammy sweat and cool surface produced by Bonwill's method. He will also understand the return of the natural status after the discontinuance of the rapid breathing.

The fact that the lungs and central organs have the blood of the body thrown upon them, awakens the careful surgeon to the danger of the indiscriminate application of the "method."

